# The Neutrophil-to-Lymphocyte Ratio (NLR) Can Predict Sepsis’s Presence and Severity in Malnourished Infants—A Single Center Experience

**DOI:** 10.3390/children10101616

**Published:** 2023-09-28

**Authors:** Alina Emilia Domnicu, Eugen Radu Boia, Mirela Mogoi, Aniko-Maria Manea, Tamara Marcela Marcovici, Otilia Mărginean, Marioara Boia

**Affiliations:** 1 Ph.D. School Department, ‘Victor Babeş’ University of Medicine and Pharmacy of Timisoara, 300041 Timisoara, Romania; mitru.alina@umft.ro; 2 Clinical Section I Pediatrics—Nutritional Recovery, Children’s Emergency Hospital ‘Louis Turcanu’, 300011 Timisoara, Romania; marcovici.tamara@umft.ro; 3 Department IX Surgery I, Discipline ENT, ‘Victor Babeş’ University of Medicine and Pharmacy of Timisoara, 300041 Timisoara, Romania; 4 ENT Department, ‘Victor Babeş’ University of Medicine and Pharmacy of Timisoara, 300041 Timisoara, Romania; 5 Pediatric Department, ‘Victor Babeş’ University of Medicine and Pharmacy of Timisoara, 300041 Timisoara, Romania; mogoi.mirela@umft.ro; 6 Neonatology and Puericulture Department, ‘Victor Babeş’ University of Medicine and Pharmacy of Timisoara, 300041 Timisoara, Romania; manea.aniko@umft.ro (A.-M.M.); boia.marioara@umft.ro (M.B.); 7 Neonatology and Preterm Department, Children’s Emergency Hospital ‘Louis Turcanu’, 300011 Timisoara, Romania; 8 Department XI Pediatrics, Discipline I Pediatrics, ‘Victor Babeş’ University of Medicine and Pharmacy of Timisoara, 300041 Timisoara, Romania; marginean.otilia@umft.ro; 9 Department of Pediatrics I, Children’s Emergency Hospital ‘Louis Turcanu’, 300011 Timisoara, Romania; 10 Department XI Pediatrics, Discipline I Pediatrics, Disturbances of Growth and Development in Children—BELIVE, 300011 Timisoara, Romania

**Keywords:** illness-related malnutrition, pediatric, sepsis, acute-phase response

## Abstract

Sepsis represents one of the leading causes of death in newborns and infants, and prompt diagnosis is essential for achieving favorable outcomes. Regarding malnourished children with concurrent infection, most studies have focused, besides blood culture, on C-reactive protein and procalcitonin. Because malnutrition has a deleterious effect on cellular immune competence, the present study characterized the acute-phase response, including hematological indices, in response to sepsis. Among the examined laboratory biomarkers, procalcitonin and neutrophil-to-lymphocyte ratio were the most accurate discriminators between sepsis patients and those with bacterial infection. Moreover, these two parameters showed a gradual increase between sepsis, severe sepsis, and septic shock patients (*p* < 0.001). Subgroup analysis of the sepsis group revealed positive correlations of NLR with prolonged ICU stay (<0.001), acute organ dysfunction (0.038), mechanical ventilation (<0.001), and fatality (<0.001). In summary, our results suggest that the neutrophil-to-lymphocyte ratio can be used as an auxiliary diagnostic index in discriminating the presence and severity of bacterial sepsis in malnourished infants.

## 1. Introduction

Despite ongoing improvements in intensive care technology and diagnostic possibilities, sepsis remains one of the most common causes of death in newborns and infants [1,2,3,4]. Early and accurate sepsis recognition is essential for favorable outcomes [5]. However, this represents a challenge, especially in infants with illness-related malnutrition, due to overlapping clinical manifestations of sepsis and preexisting chronic disease, and also a nonspecific clinical picture [6].

The World Health Organization (WHO) guidelines for the initial assessment and management of pediatric malnutrition include the diagnosis of a possible sepsis [7]. However, clinical evaluation is poorly predictive of sepsis in these patients, and microbiology services are sometimes unavailable, so other readily measurable and less costly laboratory biomarkers are needed. Although the association between pediatric malnutrition and infection has been studied for over half a century, there are still critical gaps in understanding the exact mechanisms [8].

The increased risk for developing a systemic bacterial infection in malnourished children arises from the presence of multiple factors such as prematurity, extrauterine growth restriction (EUGR), innate and adaptative immune functions compromised mainly by the protein and vitamin deficiencies, the microbial translocation across a defective gastrointestinal and respiratory mucosal barrier, and much more [9].

The acute-phase reaction in response to the stress of infection, accompanied by elevated laboratory biomarkers, such as procalcitonin (PCT), C-reactive protein (CRP), and also complete blood count (CBC)-derived indices, such as neutrophil-to-lymphocyte ratio (NLR), has been thoroughly studied over the years [10,11,12,13,14,15,16]. Other proinflammatory cytokines (TNF, IL-1, and IL-6) were also studied. Some papers reported low TNF, IL-1, and IL-6 levels in severely malnourished children consistent with the reduced febrile response in these children [17,18]. However, research regarding the acute phase response in malnourished infants with sepsis has focused mainly on PCT and CRP [17,18,19,20,21,22,23].

The data about the neutrophil function in pediatric malnutrition are scarce. It is suggested that these patients have impaired chemotaxis and lysosomal synthesis and reduced glycolytic activity, but the number is not affected [8,24,25,26,27]. The natural killer cells also have reduced activity, but the effect is reversible after the nutritional recovery [24,25,26,27]. Some descriptive studies observed defects in the adaptative immune function of these children, but for the moment, the data are incomplete and based on the vaccine response [8,24,27].

The present study aimed to characterize the response to sepsis of CBC-derived indices in infants with illness-related malnutrition, given the deleterious effect of malnutrition on cellular immune competence [24,25,26,27]. A second aim was to find a helpful biomarker for discriminating the presence and severity of sepsis in malnourished children.

## 2. Materials and Methods

### 2.1. Study Design and Patient Selection

This cross-sectional retrospective study was performed in one large Romanian reference neonatology and nutritional rehabilitation center. We reviewed 241 consecutive charts of febrile malnourished infants admitted to the Pediatric Emergency Hospital “Louis Turcanu” in Timisoara, Romania, with the suspicion of sepsis between 1 January 2017 and 31 December 2022, in accordance with the Declaration of Helsinki (1975, revised in 2013). The institutional ethics committee approved the study protocol and waived informed consent due to the study’s retrospective nature.

Patients with the following characteristics were included in the study: (1) infants ≤ 1 year of age, (2) presence of malnutrition according to WHO criteria [7], and (3) confirmed admission diagnosis of bacterial infection or sepsis. Patients with incomplete available data, those with hemato-oncological diseases, other sepsis-like presentations confirmed during and after admission (inherited metabolic disorders, intoxication, cold stress, etc.), and HIV patients were excluded.

A gestational age (GA) below 37 weeks defines a preterm birth. The measured weight and height were plotted using WHO growth charts [7] or Fenton growth charts for premature babies [28]. The z score for weight, height, and weight for height were calculated [29]. Malnutrition was defined as stunned (z score < −2.0 SDS in height for age), wasted (z score < −2.0 SDS in weight for height), or underweight (SDS <−2.0 SDS in weight for age) [7,28,29].

Patients were dichotomized into two groups: those with bacterial infection and those with sepsis. According to the International Pediatric Sepsis Consensus [30], bacterial infection was considered any clinical syndrome with a high probability of bacterial infection (positive findings on physical exam, chest radiograph with lobar consolidation or bronchopneumonia, procalcitonin ≥ 0.5 ng/mL) or infection confirmed by positive culture. Sepsis was defined as suspected or proven infection with at least two of the following systemic inflammatory response syndrome (SIRS) criteria: (1) body temperature > 38.5 °C or <36 °C, (2) mean heart rate of more than +2 standard deviations (SD) above normal for age in the absence of external stimuli, or unexplained persistent elevation; OR for children under the age of 1 year: bradycardia defined as a mean heart rate <10th percentile for age, or unexplained persistent depression over a 0.5 h, (3) mean respiratory rate of more than +2 SD above normal for age or need for mechanical ventilation; (4) leukocytosis, leukopenia, or >10% immature neutrophils. Severe sepsis refers to sepsis with cardiovascular organ dysfunction, acute respiratory distress syndrome, or ≥2 organ dysfunctions. Septic shock is considered sepsis with cardiovascular dysfunction despite isotonic fluid administration [30].

### 2.2. Clinical Assessments and Laboratory Measurements

The following data were extracted: demographic characteristics (age, gender), anthropometric measurements (body weight, height), evolution—including length of Intensive Care Unit (ICU) stay, mechanical ventilation and survival rate, primary diagnosis (infection, sepsis, septic shock) according to the definition by Goldstein et al. [30], and comorbidities. Body weight and height were measured (unclothed) with an infant scale using an attached infantometer (Seca 376 electronic baby scale; Seca Ltd., Hamburg, Germany). Laboratory analysis performed at the time of hospital admission or on the day of drawing blood culture included: CBC using an automated hematology analyzer (Sysmex XN-550, Sysmex Corporation, Kobe, Japan), CRP using a routine automated analyzer (Hitachi 747, Hitachi, Tokyo, Japan), and PCT levels using an electrochemiluminescence assay (Elecsys^®^ BRAHMS PCT kit, Roche Diagnostic, Rotkreuz, Switzerland) on a Cobas^®^ 8000 modular analyzer (Roche Diagnostic, Rotkreuz, Switzerland). Blood cultures drawn during fever were incubated in the BacT/Alert 3D automated blood culture system (BioMerieux, Craponne, France). Based on retrospectively available data, the following CBC-derived indices were calculated: NLR (neutrophil count/lymphocyte count) [31], SII (platelet count × NLR) [32], and SIRI (neutrophil count × monocyte/lymphocyte count) [33].

### 2.3. Statistical Analysis

The two study groups were defined using descriptive statistics (percentage, mean ± standard deviation (SD), median, and interquartile range (IQR)). The normality of variable data was tested using the Shapiro–Wilk test. Normally distributed continuous variables were expressed as mean ± SDs and compared using the independent *t*-test. Continuous variables with skewed distribution were plotted as medians (25th and 75th interquartile range (IQR)) and compared using the Mann–Whitney U and Kruskal–Wallis tests. Categorical variables were presented as numbers and percentages (*n*, %) and analyzed using the Chi-squared test. Spearman’s rank correlation coefficient (r) was used to investigate the relationships between NLR, PCT, and CRP and sepsis evolution. Multiple linear regression analysis was performed to verify which laboratory infection biomarkers (PCT, CRP, and NLR) are independently associated with sepsis. Ultimately, the receiver operating characteristic (ROC) curve was plotted to assess the discrimination ability of those variables in identifying septic patients. Youden’s index (calculated as sensitivity + specificity − 1) determined optimal threshold values. Statistical analyses were performed using Statistical Package for Social Sciences software (SPSS v28.0.1.1., IBM Corporation, Armonk, NY, USA). A two-sided *p* value below 0.05 was considered statistically significant.

## 3. Results

### 3.1. Patient Characteristics

A total of 167 infants with illness-related malnutrition were included in the study, of which almost two halves were septic. The clinical characteristics of the infants enrolled are summarized in Table 1. Most patients were under six months, with a mean age of 3 months for both groups. While male gender was more prevalent among both groups, the percentage was lower in the sepsis group. All infants displayed acute malnutrition, with more than half (58.6%) having multiple anthropometric deficits: 43.7% associated stunting and 34.7% underweight. The sepsis group included cases with a more severe malnutrition grade (mean z score for weight of −2.48), as opposed to the infection group (mean z score for weight of 2.09). Underweight was more prevalent among the sepsis group (*p* = 0.008).

Almost two-thirds (70.1%) of the entire study group had underlying chronic disease, with no significant statistical differences regarding associated comorbidities between infants with bacterial infection and those with sepsis.

As expected, in terms of laboratory parameters, infants with sepsis presented more elevated levels of classic infection biomarkers such as CRP and procalcitonin (*p* < 0.001). Moreover, significant statistical differences were noted between the two groups when analyzing the CBC-derived indices.

**Figure 1:**
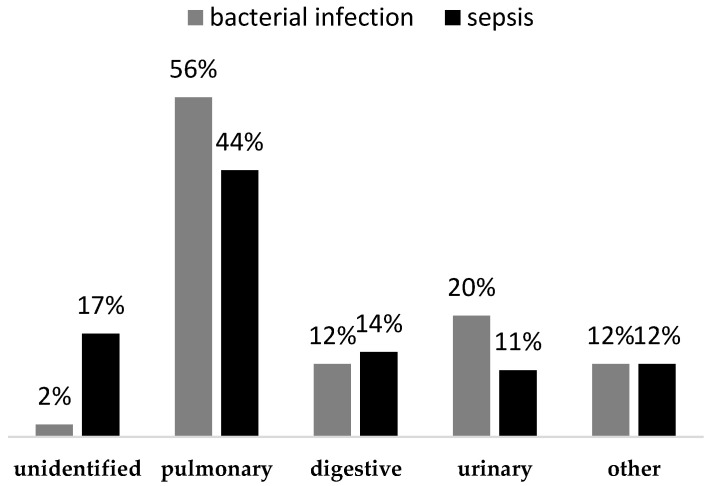
The prevalence of infection type among the entire sample.

Regarding infection source, as illustrated in Figure 1, pneumonia was the most common cause among both study groups, followed by digestive and urinary tract infections. The infection source remained unidentified in 16.9% of sepsis cases (18 patients).

### 3.2. Statistical Analysis of Clinical Characteristics of Sepsis Infants

**Figure 2:**
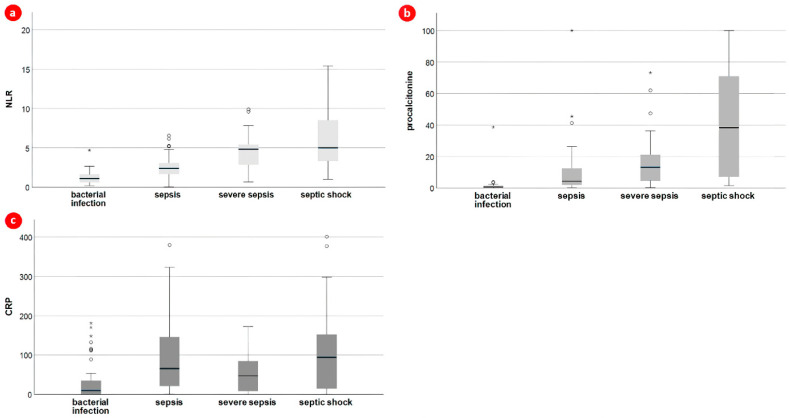
Box plot of (**a**) neutrophil-to-lymphocyte ratio, (**b**) procalcitonin, and (**c**) C-reactive protein based on presence and severity of sepsis (Kruskal–Wallis H test); * far out outliers; ◦ mild outliers.

We also performed subgroup analysis to characterize the sepsis group and to evaluate the relationship between infection biomarkers and sepsis severity, as depicted in Table 2 and Figure 2. Of the sepsis group, 27% required intubation and mechanical ventilation, and 43.3% required ICU hospitalization for more than 7 days. The overall case fatality of our sepsis group was 14.2%, with the majority (86.7%) having multiple anthropometric deficits.

Regarding infection biomarkers, only PCT and NLR showed a gradual increase between the three groups (*p* < 0.001).

### 3.3. Correlation Analysis between NLR and Clinical Variables

In order to further assess the relationship between the NLR and sepsis outcomes, we performed Spearman’s correlation analysis. As depicted in Table 3, while both NLR and procalcitonin correlate with sepsis severity, only NLR positively correlates with all investigated outcome parameters. In addition, no significant correlations were found for CRP, except for prolonged ICU stay (*p* = 0.032).

### 3.4. Diagnostic Performances of PCT, CRP, and NLR

**Figure 3:**
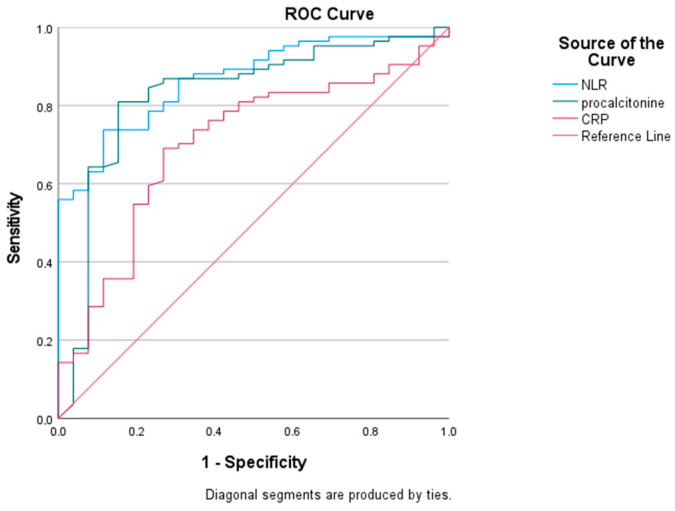
The area under the curve of neutrophil-to-lymphocyte ratio, procalcitonin, and C-reactive protein as markers of sepsis in malnourished infants.

Receiver operating characteristic (ROC) curves were plotted to assess the accuracy of PCT, CRP, and NLR in diagnosing sepsis among the entire study lot (Figure 3). The area under the ROC (curve) revealed similar excellent discriminatory power of NLR and PCT in recognizing septic cases as opposed to CRP, with NLR, PCT, and CRP having sensitivities of 0.85, 0.82, and 0.70 and specificities of 0.69, 0.76, and 0.65, respectively. The threshold values determined by Youden’s index for NLR, PCT, and CRP were 1.43, 1.56 ng/mL, and 28.3 mg/L, respectively (Table 4).

### 3.5. Association between NLR Levels and Sepsis

Logistic regression analysis was further employed to investigate the independence of NLR in predicting sepsis among our study groups, with sepsis as the dependent variable and NLR, PCT, and CRP as independent variables. After multivariable risk adjustment for age, all three infectious biomarkers remained positively associated with sepsis (Table 5).

## 4. Discussion

Our findings suggest that NLR helps identify malnourished infants with sepsis and can discriminate sepsis severity.

Illness-related malnutrition, the most common form in the developed world, generates annually increased healthcare costs due to prolonged hospitalization [34,35,36,37,38]. In addition, nutritional status further deteriorates in up to 50% of children during hospital stay [39]. Irrespective of growth restriction at birth, preterm infants are vulnerable to EUGR during neonatal stay and after discharge, related to cumulative protein and energy deficits [40]. The catch-up growth occurs mainly in the first six months of life and continues until the age of two. Because of the immaturity of the gastrointestinal tract’s immune system and the high risk for malnutrition, preterm infants are predisposed to infectious morbidity. De Sousa et al. observed that the risk of developing late-onset sepsis was 4.9-fold higher in children without appropriate catch-up growth [9]. The presented study group included a relatively high number of preterm children (*n* = 96, 57.5%). One in two infants diagnosed with sepsis was premature.

Sepsis is one of the leading causes of ICU admission and prolonged hospitalization among infants with illness-related malnutrition [41]. Therefore, prompt recognition and management are vital for favorable results [5]. Sepsis remains a major challenge because symptoms can be insidious and nonspecific [6,42,43]. The cornerstone for the pathogenesis of sepsis is inflammation, infection acting as a potent stimulus for the acute-phase response [44,45]. Therefore, research regarding the successful diagnosis of sepsis has focused, besides blood cultures, on inflammatory biomarkers [44]. With respect to nutritional status, most studies have highlighted CRP and PCT as solid markers of concurrent infection in malnourished children, similar to the acute-phase response of well-nourished children [18,46,47,48]. Regarding the prediction of sepsis severity, studies have indicated that elevated CRP and PCT levels were fair indicators of severity, being associated with fatal outcomes but not sufficiently accurate for the diagnosis of invasive bacterial infections in this specific population [20]. In contrast, Manary et al. declared lower CRP levels in infected children with malnutrition [49].

During the last decade, the research regarding sepsis has also included CBC-derived indices. A growing number of studies have pointed out that NLR can be used as a biomarker for systemic inflammatory response due to bacterial infection [50,51,52,53]. This can be explained by the fact that neutrophils increase in the context of bacterial infection through stimulation, demargination, and delayed apoptosis, while lymphocytes are decreased due to migration to the infection site and apoptosis [54,55,56,57,58,59,60,61]. Moreover, pro-inflammatory cytokines activate the anterior hypothalamus, enabling the adrenal gland to release glucocorticoids and aggravate neutrophilia [43]. Regarding the pediatric population, studies involving CBC-derived indices and bacterial sepsis have focused on well-nourished children [53]. Given the fact that, according to different authors, malnutrition has a deleterious effect on cellular immune competence [23,24,25,26,27], the present study aimed at assessing the value of CBC-derived indices in malnourished infants.

Our study group consisted mainly of infants with illness-related malnutrition, with more than half displaying multiple anthropometric deficits. As reported by other studies regarding malnutrition in developed countries, cyanotic heart lesions and gastrointestinal disease were among the most prevalent comorbidities [62,63], along with complications associated with premature birth (bronchopulmonary dysplasia, neurologic disease). In terms of infection source, pneumonia was prevalent among both study groups. This is in keeping with the findings of previous various articles that mention malnutrition’s negative effect on respiratory function by affecting the ventilator drive, causing respiratory muscle weakness, and disrupting pulmonary defense mechanisms [64,65,66,67,68,69]. The mortality rate among our entire study sample was 8.98%, with the majority (86.7%) having multiple anthropometric deficits. This is similar to the results of a previous study which stated that children with multiple anthropometric deficits are 12 times more likely to die than well-nourished children, as opposed to a two-fold increase in children who only exhibit wasting [70]. In contrast, de Souza Menezes et al. found no significant association between mortality and malnutrition in critically ill children [64].

To the best of our knowledge, this study is the first to address the NLR’s diagnostic and prognostic performances in infants with malnutrition. Among the examined laboratory biomarkers, NLR and PCT were the most accurate discriminators between sepsis patients and those with bacterial infection, with areas under the ROC curve offering ‘excellent’ discrimination, according to Hosmer and Lemeshow [71]. An NLR value of 1.43 identified subjects with sepsis with 85% sensibility and 69% specificity. Studies assessing the diagnostic value of NLR in children with sepsis have provided variable results. In terms of a relatively similar age group, this cut-off was similar to that obtained by Dursun et al. regarding a study group with a median age of 18 months that provided a threshold NLR value of 1.97 for discriminating sepsis from non-sepsis; however, the specificity and sensitivity were both lower, 75.6% and 38.4%, respectively [53]. Another study, by Goldberg et al. suggested an NLR threshold of 1.5 for diagnosing late-onset neonatal sepsis, with a sensitivity of 83.9% and a specificity of 79% [72]. Omran et al., 2018 disclosed a sensitivity of 80% and a specificity of 57.1% at a threshold value of 2.7 [73]. Differences in the study population and the testing methods used might explain variations in diagnostic accuracy among studies [74].

In terms of the sepsis group, NLR proved to be positively correlated with prolonged ICU stay (<0.001), acute organ dysfunction (0.038), mechanical ventilation (<0.001), and fatality (<0.001). The results are in agreement with previous pediatric studies, which demonstrated that NLR can predict sepsis severity and outcome [54,75].

## 5. Limitations

There are, however, limitations that need to be mentioned. (1)This is a retrospective study, susceptible to inherent bias, with almost one-third of all consecutive cases having incomplete documentation or analysis. (2) The subsample sizes were relatively small and stem from a single-center study; results may not be extrapolated to other populations. (3) It was a cross-sectional study; serial measurements might provide a more complex vision of the dynamic correlation between NLR and sepsis. (4) Other pro-inflammatory markers such as TNF, IL-1, and IL-6 were not assessed. Thus, larger prospective studies are warranted.

## 6. Conclusions and Future Directions

In summary, our results suggest that the NLR can be used as an auxiliary diagnostic index, besides PCT, in discriminating the presence and severity of bacterial sepsis in malnourished infants. An NLR value of 1.43 identified subjects with sepsis with 85% sensibility and 69% specificity. In terms of the sepsis group, NLR proved to be positively correlated with prolonged ICU stay acute organ dysfunction, mechanical ventilation, and fatality. NLR value can be used in low-income settings where other sepsis laboratory markers are not always available, but there is a high malnutrition rate among infants.

We recommend that these findings be validated by multicenter studies. Moreover, longitudinal studies that evaluate these biomarkers after nutritional recovery could provide us with some new insights into the role of malnutrition in systemic infection.

## Figures and Tables

**Table 1:** Clinical characteristics of the study lot.

Parameters	Bacterial Infection (*n* = 61)	Sepsis (*n* = 106)	*p*-Value
Age (months)	3 (2, 4)	3 (2, 5)	0.033 ^U^
Males % (*n*)	75.4 (46)	51.88 (55)	0.004 **^χ2^**
Preterm birth % (*n*)	70.49 (43)	50 (53)	0.035 ^χ2^
Anthropometric parameters			
Weight (kg), median (IQR)	3.52 (3.01, 4.13)	3.86 (3.10, 5.00)	0.082 ^U^
Height (m), median (IQR)	0.53 ± 0.04	0.57 ± 0.06	<0.001 ^U^
Weight for age (z)	−2.09 (−3.07, −1.39)	−2.48 (−3.89, −1.86)	0.011 ^U^
Height for age (z)	−0.90 (−1.51, −0.35)	−0.82 (−1.45, −0.12)	0.205 ^U^
Weight for height (z)	−1.98 (−3.01, −1.20)	−2.64 (−3.90, −1.77)	0.008 ^U^
Comorbidity % (*n*)			
Absent	31.14 (19)	29.24 (31)	0.637 ^χ2^
Genetic disorder	3.27 (2)	12.26 (13)	0.174 ^χ2^
Cardiovascular disorder	14.75 (9)	24.52 (26)	0.181 ^χ2^
Gastrointestinal disorder	16.39 (10)	22.64 (24)	0.226 ^χ2^
Neurological disorder	31.14 (19)	34.90 (37)	0.340 ^χ2^
Respiratory disorder	9.83 (6)	7.54 (8)	0.975 ^χ2^
other	22.95 (14)	19.81 (22)	0.824 ^χ2^
Laboratory parameters			
CRP (mg/L)	9.65 (0.83, 36.1)	57.2 (17.11, 137)	<0.001 ^U^
PCT (ng/mL)	0.57 (0.22, 1.47)	8.81 (2.49, 23.9)	<0.001 ^U^
WBC (×10^3^ μL)	11.88 (9.94, 15.7)	18.95 (13.77, 26.82)	<0.001 ^U^
PLT (×10^3^ μL)	436 (287, 516)	332 (169, 484)	0.004 ^U^
MPV (fL)	10.03 ± 1.02	10.04 ± 1.14	0.591 ^U^
IG % (×10^3^ μL)	0.40 (0.27, 0.92)	0.70 (0.30, 2.42)	0.075 ^U^
RDW (%)	43.8 (16.1, 49.5)	45.2 (37.7, 51.1)	0.567 ^U^
NLR	1.10 (0.62, 1.64)	3.21 (2.03, 5.27)	<0.001 ^U^
SII	473 (243, 722)	935 (429, 1757)	<0.001 ^U^
SIRI	1.85 (0.97, 3.33)	5.56 (2.96, 10.23)	<0.001 ^U^

^U^ Mann–Whitney test, ^χ2^ chi-squared. Data are expressed as mean ± standard deviation, median (interquartile range, IQR) or percentage ( %). CRP, C-reactive protein; PCT, procalcitonin; WBC, white blood cell count; PLT, thrombocytes; MPV, mean platelet volume; IG, immature granulocytes; RDW, red blood cell distribution width; NLR, neutrophil-to-lymphocyte ratio; SII, systemic immune-inflammation index; SIRI, systemic inflammation response index. Statistically significant differences are represented in bold.

**Table 2:** Subgroup analysis regarding clinical parameters of the sepsis lot.

Parameters	Sepsis (*n* = 47)	Severe Sepsis (*n* = 33)	Septic Shock/MOF (*n* = 26)	*p*-Value
Age (months)	3 (2, 5)	3 (2, 4)	3.5 (2, 5)	0.508
GA (weeks)	37 (31, 39)	38 (32, 40)	37.5 (33.5, 39)	0.633
Weight for age (z)	−2.23 (−2.77, −1.41)	−2.71 (−4.03, −1.98)	−3.45 (−4.70, −2.27)	**0.002**
Height for age (z)	−0.79 (−1.31, −0.20)	−0.73 (−1.59, −0.15)	−0.84 (−1.85, −0.37)	0.904
Weight for height (z)	−2.38 (−3.11, −1.40)	−2.83 (−3.90, −2.09)	−3.47 (−5.08, −2.25)	**0.011**
Mechanical ventilation % (n)	6.38 (3)	24.2 (8)	69.2 (18)	**<0.001**
Prolonged ICU stay % (n)	31.9 (15)	48.5 (16)	57.7 (15)	**0.032**
Irresuscitable arrest % (n)	0	0	57.7 (15)	**<0.001**
CRP (mg/L)	65.5 (20.1, 154)	47.1 (7.09, 88.5)	94.1 (35.5, 51.1)	0.293
PCT (ng/mL)	4.35 (1.83, 12.4)	13.1 (4.37, 21.5)	38.3 (6.73, 71.8)	**<0.001**
WBC (×10^3^ μL)	18.9 (13.7, 24.2)	17.9 (13.9, 29.9)	19.3 (13.5, 28)	0.877
PLT (×10^3^ μL)	336 (179, 488)	362 (204, 497)	280 (90.7, 451)	0.273
MPV (fL)	9.90 (9.10, 10.6)	9.90 (9.20, 10.95)	9.60 (9.10, 10.8)	0.820
IG (×10^3^ μL)	0.40 (0.27, 1.30)	0.70 (0.30, 2.50)	1.90 (0.40, 3.30)	0.205
RDW (%)	46.1 (37.1, 51.6)	44.5 (39.3, 53.2)	45.8 (35.5, 51.1)	0.231
NLR	2.39 (1.65, 3.10)	4.80 (2.65, 5.65)	5.02 (3.10, 8.56)	**<0.001**
SII	867 (269, 1396)	1343 (511, 2300)	1145 (344, 2725)	0.061
SIRI	4.90 (2.46, 7.79)	6.79 (3.95, 12.4)	7.05 (3.41, 14.1)	0.142

Data are expressed as median (interquartile range, IQR). GA, gestational age; CRP, C-reactive protein; PCT, procalcitonin; WBC, white blood cell count; PLT, thrombocytes; MPV, mean platelet volume; IG, immature granulocytes; RDW, red blood cell distribution width; NLR, neutrophil-to-lymphocyte ratio; SII, systemic immune-inflammation index; SIRI, systemic inflammation response index. Statistically significant differences are represented in bold.

**Table 3:** Correlation analysis of infection biomarkers with outcome in the sepsis group.

	NLR		CRP		PCT	
	*r*	*p*	*r*	*p*	*r*	*p*
Prolonged ICU stay (>7 days)	0.345	<0.001	−0.214	0.032	−0.032	0.769
Acute organ dysfunction	0.201	0.038	−0.109	0.280	−0.021	0.847
Mechanical ventilation	0.529	<0.001	−0.038	0.706	0.133	0.222
Nonresuscitable arrest	0.405	<0.001	0.001	0.992	0.109	0.319
Sepsis severity	0.470	<0.001	−0.015	0.879	0.429	<0.001

Abbreviations: NLR, neutrophile-to-lymphocyte ratio; CRP, C-reactive protein; PCT, procalcitonin; *r*, Spearman correlation.

**Table 4:** Comparison of PCT, CRP, and NLR in discriminating sepsis.

	AUC	SE	95%CI	Sensitivity	Specificity	Cut-Off	*p*-Value
PCT	0.828	0.051	0.79–0.93	0.82	0.76	1.56	<0.001
CRP	0.704	0.057	0.59–0.81	0.70	0.65	28.3	0.002
NLR	0.867	0.033	0.80–0.91	0.85	0.69	1.43	<0.001

Abbreviations: NLR, neutrophile-to-lymphocyte ratio; CRP, C-reactive protein; PCT, procalcitonin.

**Table 5:** Multivariate regression analysis regarding independent predictors of sepsis in the entire study sample.

Multivariate Analysis		
Variable	β (95% CI)	*p*-Value
PCT	0.212 (0.001–0.007)	0.016
CRP	0.238 (0–0.002)	0.020
NLR	0.274 (0.010–0.045)	0.002

Abbreviations: NLR, neutrophile-to-lymphocyte ratio; CRP, C-reactive protein; PCT, procalcitonin.

## Data Availability

Data can be made available upon reasonable request due to ethical restrictions.
